# Bradycardia after Tube Thoracostomy for Spontaneous Pneumothorax

**DOI:** 10.1155/2018/6351521

**Published:** 2018-03-19

**Authors:** Yomi Fashola, Sanjeev Kaul, Douglas Finefrock

**Affiliations:** Emergency Trauma Center, Hackensack University Medical Center, 30 Prospect Avenue, Hackensack, NJ 07601, USA

## Abstract

We present the case of an elderly patient who became bradycardic after chest tube insertion for spontaneous pneumothorax. Arrhythmia is a rare complication of tube thoracostomy. Unlike other reported cases of chest tube induced arrhythmias, the bradycardia in our patient responded to resuscitative measures without removal or repositioning of the tube. Our patient, who had COPD, presented with shortness of breath due to spontaneous pneumothorax. Moments after tube insertion, patient developed severe bradycardia that responded to Atropine. In patients requiring chest tube insertion, it is important to be prepared to provide cardiopulmonary resuscitative therapy in case the patient develops a life-threatening arrhythmia.

## 1. Introduction

Spontaneous pneumothorax can be a complication of chronic obstructive pulmonary disease. Patients with spontaneous pneumothorax usually present with pleuritic chest pain, dyspnea, tachypnea, increased work of breathing, and hypoxemia. Physical exam typically reveals decreased breath sounds with hyperresonance during percussion of the affected side of the lung. Since these patients already have poor oxygen reserve, emergent tube thoracostomy may be indicated to help prevent impending tension pneumothorax or cardiovascular collapse [1]. While there are many well documented and discussed complications secondary to tube thoracostomies, cardiovascular complications are rare [2–6]. Even rarer are reported cases of arrhythmias as a complication of tube thoracostomy [2]. We present a case of a patient who after a tube thoracostomy for spontaneous pneumothorax developed severe bradycardia that was responsive to Atropine.

## 2. Case Presentation

**Figure 1:**
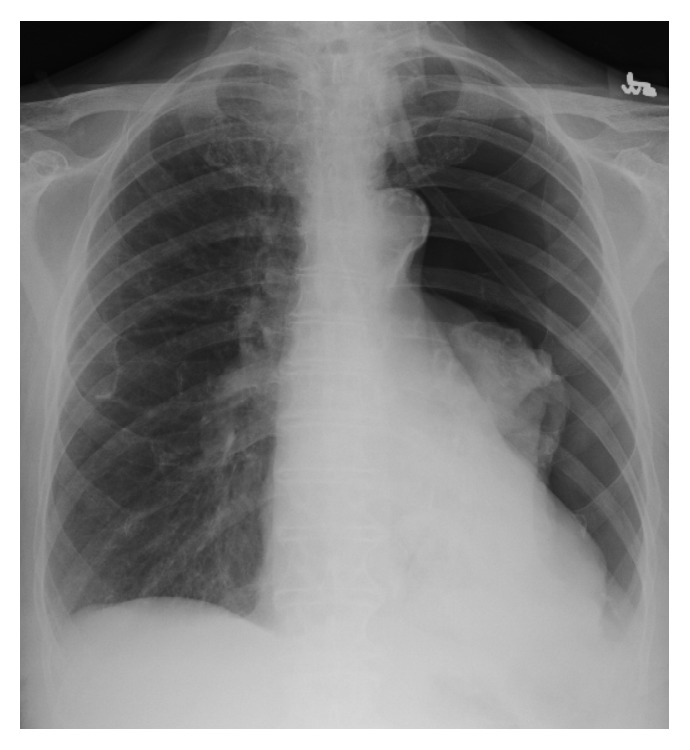
Initial portable chest X-ray showing significant left lung collapse.

**Figure 2:**
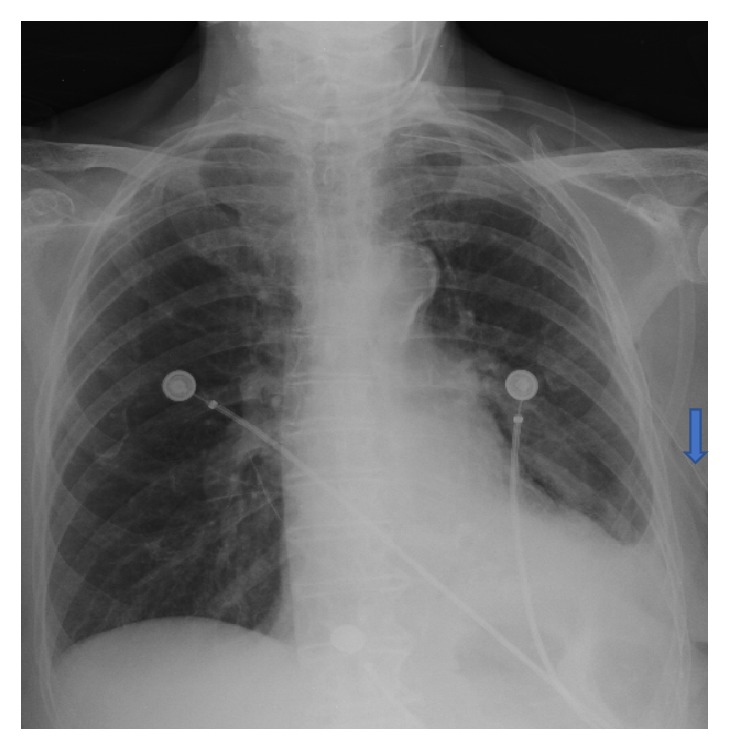
Postthoracostomy portable chest X-ray showing reexpanded left lung with residual atelectasis and or consolidation. The blue arrow points to the chest tube near the insertion point.

An 83-year-old Caucasian female with past medical history of COPD, CHF, and coronary artery disease presented to the ED for moderate persistent shortness of breath with associated chest tightness that had been going on for one day. The patient required oxygen via nasal cannula 24 hrs/day at baseline. She also had an increased productive cough. She denied pleurisy, hemoptysis, or fever. Her EKG showed sinus rhythm with right bundle branch block which was unchanged compared with her previous EKGs over the past five years. Physical exam revealed absent breath sounds as well as resonance to percussion on the left side. Suspicion of a left-sided pneumothorax was high and it was confirmed by portable chest X-ray. The chest X-ray showed a large left pneumothorax without a mediastinal shift (Figure 1). 5 mL of 2% Xylocaine was used to infiltrate the skin and a 24-Fr Argyle chest tube was promptly placed in the left 5th intercostal space at the anterior midaxillary line without any difficulty. Shortly after the tube was placed and connected to suction, the patient became severely bradycardic to 30 s. The bradycardia resolved after 0.5 mg Atropine was given and the patient's heart rate increased to 100 bpm over the course of five minutes. Position of tube was verified with postthoracostomy chest X-ray showing reexpanded left lung (Figure 2). A postthoracostomy CAT scan of the chest showed a left chest tube entering the lateral mid hemithorax, traveling superiorly and posteriorly and medially.

During her 2-week hospital stay in the intermediate care surgical unit, the patient was cared for by a multidisciplinary team and remained on telemetry. No other episodes of bradyarrhythmia or new onset arrhythmia was reported on telemetry or repeat EKGs. A transthoracic echocardiogram showed normal wall motion and no dilatation of any of the chambers. There was no pericardial disease or tamponade. She remained hemodynamically stable throughout her stay in the hospital.

## 3. Discussion

Common complications of tube thoracostomy include misplacement, injury to internal structures, infection, or tube malfunction [2]. None of these complications were encountered in our patient. While cases of cardiovascular complications secondary to chest tube insertion are rare, new onset arrhythmia shortly after chest tube placement is even rarer [2].

A case report in* The Journal of Trauma, 1994*, discussed a case of severe and fatal bradycardia in a 36-year-old patient with chest trauma shortly after tube thoracostomy. It was concluded that the tube probably caused hemorrhage which irritated the vagus nerve. This irritation resulted in refractory severe bradycardia [7]. Other case reports of postchest tube arrhythmia include cases of patients developing high-grade AV block, atrial fibrillation, or ventricular tachycardia shortly after tube insertion [8]. In each of those cases, only withdrawal of the tubes yielded a resolution of the arrhythmias. Literature review has shown that the most common arrhythmia associated with spontaneous pneumothorax is sinus tachycardia [8, 9].

Our patient had some risk factors that predisposed her to bradycardia. Her history of coronary artery disease predisposes her to acute ischemia involving tissues supplied by the right coronary artery (RCA). However, the patient had an unchanged EKG and troponin <0.03 and had normal wall motions on echocardiogram. Another risk factor present in our patient was hyperkalemia. She was hyperkalemic (5.5) at presentation. This is also unlikely to be the cause of her acute bradycardia because a more dramatic elevation of potassium (>7.0) is usually required to cause acute bradycardia when there are no other AV blockers in the picture [10–12]. She has no history of right ventricular hypertrophy, rheumatic heart disease, cor pulmonale, pulmonary embolism, or myocarditis. Finally, her history of hypothyroidism, which is another risk factor for bradycardia, is well managed with Synthroid.

It is our opinion that the sudden onset severe bradycardia in our patient shortly after chest tube placement was most likely due to either a vagal response to pain or direct stimulation of a branch of the left vagus nerve within the thorax. Pain and emotional distress from the chest tube insertion likely caused direct hypothalamic activation of the parasympathetic pathway of the cardiovascular center in the medulla oblongata. This caused increased frequency of cardioinhibitory impulses via the vagus nerve to the SA node of the heart leading to decreased heart rate [13, 14]. It is also possible that the 24 Fr tube touched a branch of the left vagus nerve, causing the bradycardia which then quickly resolved after the tube was slightly displaced by the expanding lung. In either case, Atropine is an appropriate initial therapy. If prolonged irritation or significant injury of the vagus nerve was the cause of severe bradycardia in this patient, the arrhythmia would not have simply resolved after one dose of 0.5 mg Atropine [7]. Withdrawal of the tube may have been required. It is also unlikely that the pneumothorax itself compressed the vagus nerve to cause the bradycardia because the arrhythmia started after chest tube placement.

## 4. Conclusion

While arrhythmia shortly after chest tube thoracostomy should raise suspicion of direct or indirect irritation or trauma to intrathoracic structures, the contribution of cardioinhibitory signals from vasovagal response triggered pain should also be considered. Our case differs from other case reports because our patient's bradycardia was not refractory to resuscitative measures and we did not have to remove or reposition the chest tube to achieve resolution of the arrhythmia. This case report further emphasizes the need for emergency room physicians and surgeons to be prepared to provide standard cardiovascular resuscitative interventions in the setting of chest tube thoracostomy while taking precautions to avoid injury to intrathoracic structures such as the vagus nerve.
